# Supplementary material for: Association between HIV and treatment-resistant hypertension in Malawian adults: a protocol for a case–control study
Source: BMJ Open. 2023 Aug 2;13(8):e069280. doi: 10.1136/bmjopen-2022-069280 (PMC10401204; doi:10.1136/bmjopen-2022-069280)
Supplement: Author's manuscript [file bmjopen-2022-069280.draft_revisions.pdf]

BMJ Open is committed to open peer review. As part of this commitment we make the peer review history of every article we publish publicly available.

When an article is published we post the peer reviewers' comments and the authors' responses online. We also post the versions of the paper that were used during peer review. These are the versions that the peer review comments apply to.

The versions of the paper that follow are the versions that were submitted during the peer review process. They are not the versions of record or the final published versions. They should not be cited or distributed as the published version of this manuscript.

BMJ Open is an open access journal and the full, final, typeset and author-corrected version of record of the manuscript is available on our site with no access controls, subscription charges or pay-per-view fees (http://bmjopen.bmj.com).

If you have any questions on BMJ Open's open peer review process please email info.bmjopen@bmj.com

BMJ Open

Association between HIV and treatment-resistant hypertension in Malawian adults: a protocol for a case-control study

Journal:	*BMJ Open*
Manuscript ID	bmjopen-2022-069280
Article Type:	Protocol
Date Submitted by the Author:	15-Oct-2022

Complete List of Authors:	Gondwe, Josephine; Malawi-Liverpool-Wellcome Research Programme<br>Ndovie, Maclean; Queen Elizabeth Central Hospital, Department of Medicine<br>Khuluza, Felix; Kamuzu University of Health Sciences, Department of Pharmacy<br>Banda, Clifford; Malawi-Liverpool-Wellcome Research Programme

Keywords:	CLINICAL PHARMACOLOGY, Hypertension < CARDIOLOGY, HIV & AIDS < INFECTIOUS DISEASES

SCHOLARONE™ Manuscripts

Association between HIV and treatment-resistant hypertension in Malawian adults: a protocol for a case-control study

Authors:

Josephine Gondwe[1], Maclean Ndovie[2], Felix Khuluza[3], Clifford G Banda[#1]

Affiliations:

1. Malawi-Liverpool-Wellcome Research Programme, Blantyre, Malawi
2. Department of Medicine, Queen Elizabeth Central Hospital, Blantyre, Malawi
3. Department of Pharmacy, Kamuzu University of Health Sciences, Blantyre, Malawi

Corresponding author

[#]Clifford G Banda, Malawi-Liverpool-Wellcome Research Programme, P.O.Box 30096, Blantyre, Malawi, cgbanda@mlw.mw

For peer review only

ABSTRACT

Introduction

Treatment-resistant hypertension (RH); defined as uncontrolled blood pressure (≥140/90 mm Hg) despite treatment with ≥3 medications of different classes (including diuretics) at optimal doses; is associated with poor prognosis and an elevated risk of end-organ damage. In areas where HIV is endemic, such as sub-Saharan Africa, the risk of hypertension is high in people living with HIV. It remains unknown if HIV infection further increases the risk of RH. This study seeks to determine the association between HIV and RH as well as investigate other factors associated with RH in hypertensive Malawian adults.

Methods and analysis

A case-control study will be conducted among adult hypertensive patients attending a clinic at a referral hospital in Malawi. The cases will be hypertensive patients with a confirmed diagnosis of RH. For each case, 2 controls (hypertensive patients without RH), frequency matched for age group and sex, will be selected from among hospital clients attending the same hypertension clinic as the case. In both groups, the prevalence of HIV will be ascertained. Additionally, information on other potential risk factors of RH, such as chronic kidney disease, obesity, hypercholesteremia, diabetes, smoking, alcohol use, ART regimen and duration, will be collected in both cases and controls. For each of the potential risk factors, odds ratios will be calculated to quantify the strength of their association with RH. In a multivariate analysis, conditional logistic regression will be used to assess the independent

For peer review only

association between HIV and RH as well as the influence of the other potential drivers of RH.

Ethics and dissemination

This protocol has been approved by the College of Medicine Research Ethics Committee (COMREC) in Malawi (P.05/22/3637). Findings from this study will be disseminated through a peer-reviewed publication in an open-access international journal. Furthermore, anonymised data will be available upon request from the authors.

Keywords

Hypertension, HIV, Clinical Pharmacology

ARTICLE SUMMARY

Strengths and limitations of this study

- Strength: The case-control design will allow the investigation of multiple exposures that could drive treatment-resistant hypertension in people living with HIV.
- Limitation: The study will only recruit participants from one large hypertension clinic at a referral public hospital. This has the potential to exclude other adults that could be otherwise eligible (e.g., those from private hospitals or inpatients) leading to selection bias.

For peer review only

INTRODUCTION

Over the last twenty years, sub-Saharan Africa (SSA) has faced a rise in the prevalence of non-communicable diseases (NCDs) because of an increase in cardiovascular risk factors such as poor diet, little to no physical activity, hypertension, obesity, diabetes, and dyslipidaemia(1). This rise presents a double burden on SSA since the region is also faced with a high prevalence of infectious diseases like tuberculosis, malaria, and HIV. Previous studies have shown a growing intersection between infectious diseases and NCDs, e.g., an increased prevalence of hypertension among people living with HIV (2,3). In this subgroup, several pathophysiological processes have been implicated in the causation of hypertension. These include microbial gut translocation, elevated inflammatory markers like interleukin-6 (4), antiretroviral therapy (ART)-related inflammation due to protease inhibitors, immune reconstitution syndrome, HIV- related renal disease and lipodystrophy; caused by both HIV and ART use (2,5).

Despite its high global burden, most people who suffer from hypertension, including those living with HIV, fail to achieve adequate blood pressure control and are usually unaware of their disease status, until faced with end-organ disease. In SSA, only 27% of people with hypertension are aware of their disease, 18% are receiving treatment and yet only 7% of those suffering from hypertension have well-controlled blood pressure (6). Inadequate blood pressure control may be due to several factors, including non-adherence by physicians to treatment guidelines, pharmacological treatment noncompliance, lack of physical activity or the presence of white-coat

For peer review only

hypertension (7). Treatment-resistant hypertension (RH) is another increasingly recognized cause of uncontrolled blood pressure that occurs in individuals who are adhering to optimised treatment regimens of antihypertensive medications (3).

RH is defined as uncontrolled blood pressure (≥140/90 mm Hg) despite treatment with ≥3 medications of different classes (including diuretics) at optimal doses. RH is associated with poor prognosis and elevated risk of end-organ damage which can lead to premature deaths. The global burden of RH has been estimated to be around 10.3% (3), and previous studies have estimated the burden of RH in Africa to be ~12% (8). Additionally, RH is high among obese individuals, older people (particularly >75 years), and those with chronic conditions such as chronic kidney disease, diabetes mellitus, hypercholesteremia, heart failure and stroke (7–9). However, given the geographic intersection between HIV and hypertension, there is a paucity of evidence on whether HIV infection increases the risk of RH.

Malawi, like most SSA countries, has a high burden of HIV; 10.6% (10); and a high prevalence of hypertension; ~16% (11). In this study, we hypothesise that HIV increases the risk of RH in hypertensive Malawian adult patients. Thus, we aim to determine the independent association between HIV infection and RH. Additionally, we will investigate the impact of other potential drivers of RH such as hypercholesteremia, obesity, chronic kidney disease, diabetes, ART regimen and duration, alcohol use as well as smoking. Understanding whether HIV increases the risk of treatment-resistant hypertension, and the influence of other potential drivers of

For peer review only

RH is vital in targeting early optimal preventive and therapeutic interventions in this vulnerable sub-population.

METHODS AND ANALYSIS

Study design and setting

A case-control study will be conducted at the outpatient hypertension clinic at Queen Elizabeth Central Hospital (QECH) in Blantyre, Malawi. QECH is the largest tertiary hospital in Malawi that receives referrals from across the country. It is a free public hospital hence it serves mainly the low to middle-income class. A case-control study design has been chosen for three reasons. Firstly, treatment-resistant hypertension is a relatively rare disease, and a case-control study will allow research on this outcome. Secondly, it will reduce the time constraint that a prospective cohort study would present due to the need for a longer follow-up period. Lastly. It will allow the investigation of multiple factors associated with RH.

Identifying cases and controls

Cases will be defined as hypertensive patients with a confirmed diagnosis of treatment-resistant hypertension. In this study, treatment-resistant hypertension will be defined as a blood pressure of ≥ 140/90mmHg measured using an ambulatory blood pressure monitor over 4 hours in patients with hypertension and who are on at least 3 different medications including a diuretic. In previous studies, 4-hour ambulatory blood pressure monitoring, instead of 24 hours, has been validated as an accurate proxy of day-time blood pressure (12). Controls will be defined as hypertensive patients with well-controlled blood pressure (BP <140/90mmHg)

For peer review only

regardless of how many antihypertensive medications they are currently taking. For each case, two controls, frequency matched for age group and sex, will be selected from among hospital clients attending the same hypertension clinic as the case (i.e., from the population giving rise to the case).

Recruitment

Patients who are 18 years and above presenting to the hypertension clinic at Queen Elizabeth Central Hospital will be considered for recruitment into the study. The selection of cases and controls will be conducted as outlined in figure 1 below. Simple random sampling will be employed as a sampling technique.

Suspected RH cases will be patients who have elevated blood pressure of ≥140/90mmHg on the day of recruitment and who are taking any three classes of antihypertensive medication including a diuretic. To exclude pseudo-RH (defined as elevated blood pressure ≥140/90mmHg on at least 3 medications including a diuretic, but where there is evidence of white coat hypertension as confirmed by a 24-hour ambulatory BP measurement (ABPM), or where the cause of the uncontrolled blood pressure is known (9), investigators will assess if the dosing of medications is correct and drug combination is not sub-optimal using the Malawi Standard Treatment Guidelines. Additionally, adherence to medications will be assessed through the Medication Adherence Scale (13). Thereafter, those with correct doses and optimum drug combinations, who are adherent to their medication will be assessed for white coat hypertension (or lack thereof) by taking blood pressure measurements continuously over 4 hours using an ambulatory blood

For peer review only

pressure monitor. If a patient still has a blood pressure reading of ≥140/90mmHg after this time, they will be defined as having resistant hypertension and will make up the case group for this study.

The controls will be sex and age-group-matched individuals who have well-controlled blood pressure (Bp<140/90mmHg). After obtaining consent in both groups, HIV status will be ascertained. Other factors such as chronic kidney disease, obesity, hypercholesteremia, diabetes, smoking, alcohol use, ART regimen and duration will be assessed through the collection of medical history, review of notes in health passport books, and blood tests.

Inclusion criteria for cases

- Non-pregnant adults (≥18 years)
- Patients taking at least 3 classes of antihypertensive medication including a diuretic for any length of time.
- Patients with high blood pressure readings of ≥ 140/90mmHg on the last 2 hospital visits.
- Patients with a blood pressure reading of ≥ 140/90mmHg on the date of enrollment.

Exclusion criteria for cases

- Patients over 18 years with blood pressure readings of ≥ 140/90mmHg who are taking no more than two classes of antihypertensive medication.

For peer review only

- Patients taking at least 3 classes of antihypertensive medication but not including a diuretic on their drug regimen.

Inclusion criteria for controls

- Non-pregnant adults (≥18 years) who have well-controlled blood pressure (BP < 140/90mmHg) taking any classes of antihypertensive medication.
- Patients must have had well-controlled blood pressure (BP<140/90mmHg) on their last 2 hospital visits.

Exclusion criteria for controls

- Patients over 18 years with blood pressure readings of ≥140/90mmHg
- Patients over 18 years with well-controlled blood pressure (BP<140/90mmHg), who are not taking any antihypertensive medication.

Data collection

Details of the data to be that will be collected are outlined in Table 1. The research staff will first collect demographic data from all participants. Thereafter, participants will be asked about their medical and social history. For this study, information on previous diagnoses of hypertension, HIV, kidney disease, diabetes and high cholesterol will be collected. Information including duration of illness and dose of medication will also be sought. The investigators will measure the blood pressure of both cases and controls using a digital blood pressure cuff. After 30 minutes of the participants' rest and whilst the participant is seated, the investigators will collect 3

For peer review only

blood pressure readings 5 minutes apart. This method was used in previous studies to measure blood pressure in Malawian adults(11). For participants who have pseudo-resistant hypertension, an ambulatory blood pressure monitor will be used (as illustrated in figure 1 below). Thereafter, blood pressure will be measured using ambulatory blood pressure monitoring (ABPM) cuffs over 4 hours. The 4-hour ABPM will be adopted in this study since previous studies have shown that a 4-hour ABPM window can be used to accurately represent daytime blood pressure (12). Lastly, body mass index (BMI) will be calculated and categorized by age, sex and percentile.

Objective 1: ***Assessing the association between HIV infection and RH***

In a private environment, participants will first be asked if they are aware of their HIV status. HIV status will be deemed positive if the individual is on ART or has a documented positive result in their health passport. Those who have been taking ART will provide more information on regimen(s) and how long they have been taking the drugs. This includes asking if they have been switched on ART previously.

Individuals with documented negative HIV test result in their health passports within the last three months will be marked as negative. Three months have been employed in tandem with the window period for HIV infection. Rapid antibody tests (which is what will be used in this study) can accurately detect HIV infection 90 days after exposure. An HIV test will be offered after relevant informed consent and pre-test counselling to those who are uncertain about their status or who verbally indicate that they have a negative result without documentation in their health

For peer review only

passport (but are willing to have their HIV status confirmed). Post-test counselling will be offered, and if found positive, participants will be referred for ART initiation. The HIV test will be done by trained providers at the Accidents and Emergency department at QECH.

Objective 2: *Identifying risk factors or predictors of RH*

In previous studies (7,8,14), it has been shown that clinical conditions including chronic kidney disease, obesity and dyslipidaemia are associated with a higher risk of RH. In this study, these three conditions will be studied as (modifiable) risk factors. Screening for the presence of these comorbidities is part of the standard of care in patients in hypertensive patients. In circumstances where recent (<3 months) blood results to monitor for these conditions are not available, blood samples will be collected to conduct two biochemistry tests: creatinine and total cholesterol from both cases and controls. 5mls of blood will be drawn in a red top test tube to conduct the aforementioned tests. These tests will be conducted at the Malawi-Liverpool-Wellcome Research Programme's laboratory. Additionally, the weight and height of clients will be measured to calculate the body mass index. Furthermore, information on diabetes, smoking, alcohol use, ART regimen and duration (for those living with HIV) will be collected through participant interviews and from hospital records.

Primary outcome

The primary outcome of the study is the adjusted odds ratio, with a 95% confidence interval, of the association between RH and HIV. This will be defined as the odds of HIV in RH patients divided by the odds of HIV in hypertensive patients without RH.

For peer review only

249

250 Secondary outcomes

251 The secondary outcome measures are the odds ratios, with 95% confidence
252 intervals, of the independent association between the covariates representing other
253 potential risk factors; cholesterol, obesity, kidney disease; and RH.

254

255 Sample size

256 In calculating the sample size, a three-fold relative difference in the odds of HIV
257 infection in cases compared with controls was considered clinically significant.
258 Assuming an HIV prevalence of 10.6% among controls, as is seen in the general
259 population (10), a sample size of 66 cases and 132 controls (a total of 198) will
260 provide at least 80% power, at a 5% level of significance, to detect a three-fold
261 relative difference in the odds of exposure (HIV infection) between those with- and
262 without RH (i.e., the odds ratio of HIV infection in cases relative to controls = 3).
263 These computations were conducted using STATA 17, using the function of
264 *estimating sample size for a matched case-control study*

265

266 Data management

267 All data in the study will be collected and managed using the Open-Data Kit software
268 platform. Data will be entered by the study team, and the data accuracy will be
269 verified by the study principal investigator (PI). Only study team members will have
270 access to protected health information. All study-related information will be stored
271 securely at the study site. Tablet computers used to collect the data will be password

For peer review only

protected. Any physical participant information will be stored in locked file cabinets in areas with limited access. Patients will not be identified by name in any reports on this study. The study PI and co-investigators will have access to the final dataset of the study.

Data analysis plan

The number and percentages of cases and controls identified, and those recruited into the study and participants included in the final analysis will be reported. Additionally, the median (range) of baseline continuous variables (age, weight, height, body mass index, blood pressure, total cholesterol, and creatinine values in both groups) will be described for both cases and controls. Furthermore, the prevalence of other risk factors; HIV, obesity, hypercholesteremia and chronic kidney disease in both RH cases and non-RH controls, will be described. In participants living with HIV, different ART regimens and documented viral load and/or CD4 counts, in both controls and cases, will be compared.

To determine the primary and secondary outcomes, two main steps will be carried out. Firstly, the crude odds ratios of the association between RH and exposure characteristics of interest: HIV, obesity, chronic kidney disease, and hypercholesteremia; will be computed in univariate analysis with conditional logistic regression models. Similarly, the relationships between other exposure variables; smoking, alcohol intake and diabetes; and RH will be established. In participants living with HIV, we will explore the relationship between the different ART regimens and RH. Statistical significance will be assessed at a 5% level of significance. Except

For peer review only

296 for HIV, only covariates that show statistical significance in this univariate analysis
297 will be carried on to the next step.

298

299 Secondly, using a backward stepwise approach in a conditional logistic regression
300 model, the adjusted association between RH and HIV will be ascertained. Covariates
301 to be adjusted for will be those that show a crude statistically significant association
302 with RH in the first step above. For all analyses, the $\chi 2$ test will be used to compare
303 categorical data between cases and controls, while the Wilcoxon Rank Sum test will
304 be used for continuous data. All analyses will be conducted using STATA 17
305 software.

306

307 **Patient and public involvement statement**

308 Patients and the public were not involved in setting the research priorities, defining
309 the research question, or determining the outcomes of this study.

310

311 **ETHICS AND DISSEMINATION**

312 This study has been approved by the College of Medicine Research Ethics
313 Committee (COMREC) with approval number P.05/22/3637. Written consent will be
314 sought from participants before enrolment into the study. Findings from this study will
315 be disseminated through submission for publication in an international, open-access,
316 peer-reviewed journal. Anonymised data from the study will be made available upon
317 request from the authors.

For peer review only

REFERENCES

1. Bigna JJ, Noubiap JJ. The rising burden of non-communicable diseases in sub-Saharan Africa. Vol. 7, The Lancet Global Health. Elsevier Ltd; 2019. p. e1295–6.

2. van Zoest RA, van den Born BJH, Reiss P. Hypertension in people living with HIV. Vol. 12, Current Opinion in HIV and AIDS. Lippincott Williams and Wilkins; 2017. p. 513–22.

3. Xu Y, Chen X, Wang K. Global prevalence of hypertension among people living with HIV: a systematic review and meta-analysis. Vol. 11, Journal of the American Society of Hypertension. Elsevier Ireland Ltd; 2017. p. 530–40.

4. Tenorio AR, Zheng Y, Bosch RJ, Krishnan S, Rodriguez B, Hunt PW, et al. Soluble markers of inflammation and coagulation but not T-cell activation predict non-AIDS-defining morbid events during suppressive antiretroviral treatment. Journal of Infectious Diseases. 2014 Oct 15;210(8):1248–59.

5. Fahme SA, Bloomfield GS, Peck R. Hypertension in HIV-infected adults: Novel pathophysiologic mechanisms. Vol. 72, Hypertension. Lippincott Williams and Wilkins; 2018. p. 44–55.

6. Ataklte F, Erqou S, Kaptoge S, Taye B, Echouffo-Tcheugui JB. The burden of undiagnosed hypertension in sub-Saharan Africa: a systematic review and meta-analysis. Hypertension [Internet]. 2014;16. Available from: http://hyper.ahajournals.org/content/early/2014/11/10/HYPERTENSIONAHA.114.04394http://hyper.ahajournals.org/content/suppl/2014/11/10/HYPERTENSIONAHA.114.04394.DC1.htmlDataSupplement

For peer review only

7. Myat A, Redwood SR, Qureshi AC, Spertus Director JA, Williams B. Resistant hypertension. Vol. 345, BMJ (Online). 2012.

8. Nansseu JRN, Noubiap JJN, Mengnjo MK, Aminde LN, Essouma M, Jingi AM, et al. The highly neglected burden of resistant hypertension in Africa: a systematic review and meta-analysis. BMJ Open. 2016 Sep 20;6(9):e011452.

9. Noubiap JJ, Nansseu JR, Nyaga UF, Sime PS, Francis I, Bigna JJ. The global prevalence of resistant hypertension: A meta-analysis of data from 3.2 million patients. Vol. 105, Heart. BMJ Publishing Group; 2019. p. 98–105.

10. Malawi Population-based HIV Impact Assessment (MPHIA [Internet]. Available from: http://phia.icap.columbia.edu

11. Price AJ, Crampin AC, Amberbir A, Kayuni-Chihana N, Musicha C, Tafatatha T, et al. Prevalence of obesity, hypertension, and diabetes, and cascade of care in sub-Saharan Africa: a cross-sectional, population-based study in rural and urban Malawi. Lancet Diabetes Endocrinol. 2018 Mar 1;6(3):208–22.

12. Chanudet Xavier, Chau Phong Nguyen, Larroque Pierre. Short-term representatives of daytime and night-time ambulatory blood pressures. J Hypertens. 1992 Jun;10(6):595–600.

13. Morisky DE, Ang A, Krousel-Wood M, Ward HJ. Predictive validity of a medication adherence measure in an outpatient setting. J Clin Hypertens [Internet]. 2008 May 2 [cited 2022 Oct 9];10(5):348–54. Available from: https://onlinelibrary.wiley.com/doi/full/10.1111/j.1751-7176.2008.07572.x

14. Lotufo PA, Pereira AC, Vasconcellos PS, Santos IS, Mill JG, Bensenor IM. Resistant hypertension: Risk factors, subclinical atherosclerosis, and

For peer review only

comorbidities among adults-the Brazilian longitudinal study of adult health (ELSA-Brasil). J Clin Hypertens. 2015 Jan 1;17(1):74–80.

For peer review only

TABLES

Table 1. Details of data to be collected

	Enrolment
**Baseline characteristics**	
Name	X
Age	X
Sex	X
Phone Number	X
**Social history**	
Alcohol intake	X
Smoking	X
**Medical history**	
HIV	X
Hypertension	X
Kidney disease	X
Hypercholesteremia	X
Diabetes	X
Other medical diagnoses	X
**Anthropometric measurements**	
Height	X
Weight	X
**Blood pressure measurement**	
Systolic blood pressure	X
Diastolic blood pressure	X
4-hour ambulatory blood pressure monitoring	X
**Blood results**	
Creatinine	X
Total cholesterol	X
HIV rapid test	X

For peer review only

FIGURE AND TABLE LEGENDS

Table 1: Details of data to be collected

Figure 1. Description of study design and profile

AUTHORS STATEMENT.

JG and CGB conceptualized the research question. JG drafted the protocol manuscript. MN, FK and CGB reviewed the protocol manuscript. JG and MN will have oversight of data collection with supervision from FK and CGB.

FUNDING

This work will be supported through a Pre-MSc Fellowship Grant to JG; grant number WTT803/JG; awarded by the Joint Kamuzu University of Health Sciences and the Malawi-Liverpool-Wellcome Research Programme Training Committee with Core Training Support from the Wellcome.

CONFLICT OF INTERESTS

None

WORD COUNT

2,523

For peer review only

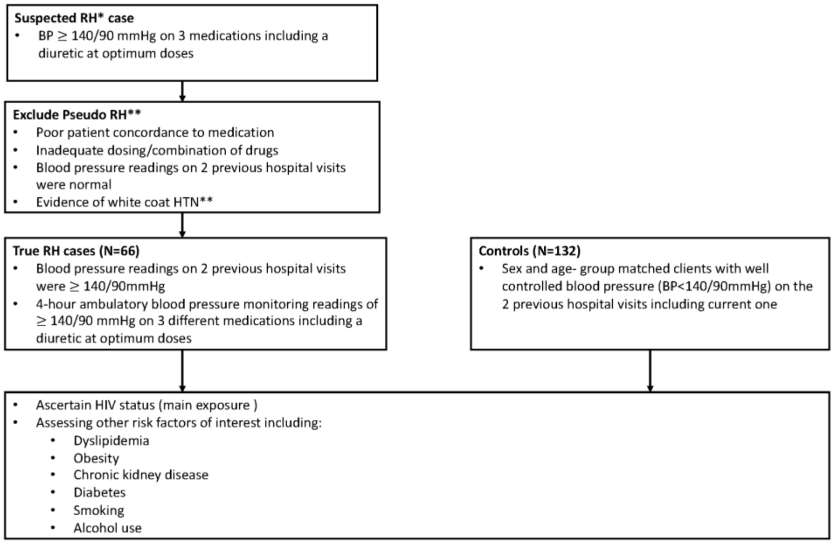

Figure 1. Description of study design and profile.
* Treatment-resistant hypertension
** Hypertension

338x190mm (200 x 200 DPI)

BMJ Open

Association between HIV and treatment-resistant hypertension in Malawian adults: a protocol for a case-control study

Journal:	*BMJ Open*
Manuscript ID	bmjopen-2022-069280.R1
Article Type:	Protocol
Date Submitted by the Author:	31-Mar-2023

Complete List of Authors:	Gondwe, Josephine; Malawi-Liverpool-Wellcome Research Programme<br>Ndovie, Maclean; Queen Elizabeth Central Hospital, Department of Medicine<br>Khuluza, Felix; Kamuzu University of Health Sciences, Department of Pharmacy<br>Banda, Clifford; Malawi-Liverpool-Wellcome Research Programme

**Primary Subject Heading**:	Pharmacology and therapeutics
Secondary Subject Heading:	Cardiovascular medicine, Medical management
Keywords:	CLINICAL PHARMACOLOGY, Hypertension < CARDIOLOGY, HIV & AIDS < INFECTIOUS DISEASES

SCHOLARONE™ Manuscripts

Association between HIV and treatment-resistant hypertension in Malawian adults: a protocol for a case-control study

Authors:

Josephine Gondwe[1], Maclean Ndovie[2], Felix Khuluza[3], Clifford G Banda[#1]

Affiliations:

1. Malawi-Liverpool-Wellcome Research Programme, Blantyre, Malawi
2. Department of Medicine, Queen Elizabeth Central Hospital, Blantyre, Malawi
3. Department of Pharmacy, Kamuzu University of Health Sciences, Blantyre, Malawi

Corresponding author

[#]Clifford G Banda, Malawi-Liverpool-Wellcome Research Programme, P.O.Box 30096, Blantyre, Malawi, cgbanda@mlw.mw

For peer review only

ABSTRACT

Introduction

Treatment-resistant hypertension (RH); defined as uncontrolled blood pressure (≥140/90 mm Hg) despite treatment with ≥3 medications of different classes (including diuretics) at optimal doses; is associated with poor prognosis and an elevated risk of end-organ damage. In areas where HIV is endemic, such as sub-Saharan Africa, the risk of hypertension is high in people living with HIV. It remains unknown if HIV infection further increases the risk of RH. This study seeks to determine the association between HIV and RH as well as investigate other factors associated with RH in hypertensive Malawian adults.

Methods and analysis

A case-control study will be conducted among adult hypertensive patients attending a clinic at a referral hospital in Malawi. The cases will be hypertensive patients with a confirmed diagnosis of RH. For each case, 2 controls (hypertensive patients without RH), frequency matched for age group and sex, will be selected from among hospital clients attending the same hypertension clinic as the case. In both groups, HIV status will be ascertained. Additionally, information on other potential risk factors of RH, such as chronic kidney disease, obesity, hypercholesteremia, diabetes, smoking, alcohol use, ART regimen and duration, will be collected in both cases and controls. For each of the potential risk factors, odds ratios will be calculated to quantify the strength of their association with RH. In a multivariate analysis,

For peer review only

conditional logistic regression will be used to assess the independent association between HIV and RH as well as the influence of the other potential drivers of RH.

Ethics and dissemination

This protocol has been approved by the College of Medicine Research Ethics Committee (COMREC) in Malawi (P.05/22/3637). Findings from this study will be disseminated through a peer-reviewed publication in an open-access international journal. Furthermore, anonymised data will be available upon request from the authors.

Keywords

Hypertension, HIV, Clinical Pharmacology

ARTICLE SUMMARY

Strengths and limitations of this study

- A key strength of the case-control design is that it will allow the investigation of multiple exposures that could drive treatment-resistant hypertension in people living with HIV.
- To our knowledge, this is the first study in Malawi to understand drivers of treatment-resistant hypertension and will generate important knowledge to inform future cohort studies in settings where HIV is highly prevalent.

For peer review only

- The study will only recruit participants from one large hypertension clinic at a referral public hospital. This has the potential to exclude other adults that could be otherwise eligible (e.g., those from private hospitals or inpatients) leading to selection bias.

For peer review only

INTRODUCTION

Over the last twenty years, sub-Saharan Africa (SSA) has faced a rise in the prevalence of non-communicable diseases (NCDs) because of an increase in cardiovascular risk factors such as poor diet, little to no physical activity, hypertension, obesity, diabetes, and dyslipidaemia.[1] This rise presents a double burden on SSA since the region is also faced with a high prevalence of infectious diseases like tuberculosis, malaria, and HIV. Previous studies have shown a growing intersection between infectious diseases and NCDs, e.g., an increased prevalence of hypertension among people living with HIV.[2,3] In this subgroup, several pathophysiological processes have been implicated in the causation of hypertension. These include microbial gut translocation, elevated inflammatory markers like interleukin-6,[4] antiretroviral therapy (ART)-related inflammation due to protease inhibitors, immune reconstitution syndrome, HIV- related renal disease and lipodystrophy; caused by both HIV and ART use.[2,5]

Despite its high global burden, most people who suffer from hypertension, including those living with HIV, fail to achieve adequate blood pressure control and are usually unaware of their disease status, until faced with end-organ disease. In SSA, only 27% of people with hypertension are aware of their disease, 18% are receiving treatment and yet only 7% of those suffering from hypertension have well-controlled blood pressure.[6] Inadequate blood pressure control may be due to several factors, including non-adherence by physicians to treatment guidelines, pharmacological treatment noncompliance, lack of physical activity or the presence of white-coat hypertension.[7] Treatment-resistant hypertension (RH) is another increasingly

For peer review only

recognized cause of uncontrolled blood pressure that occurs in individuals who are adhering to optimised treatment regimens of antihypertensive medications.[3]

RH is defined as uncontrolled blood pressure (≥140/90 mm Hg) despite treatment with ≥3 medications of different classes (including diuretics) at optimal doses. RH is associated with poor prognosis and elevated risk of end-organ damage which can lead to premature deaths. The global burden of RH has been estimated to be around 10.3%,[3] and previous studies have estimated the burden of RH in Africa to be ~12%.[8] Additionally, RH is high among obese individuals, older people (particularly >75 years), and those with chronic conditions such as chronic kidney disease, diabetes mellitus, hypercholesteremia, heart failure and stroke.[7–9] However, given the geographic intersection between HIV and hypertension, there is a paucity of evidence on whether HIV infection increases the risk of RH.

Malawi, like most SSA countries, has a high burden of HIV; 10.6% [10] and a high prevalence of hypertension; ~16%.[11] In this study, we hypothesise that HIV increases the risk of RH in hypertensive Malawian adult patients. Thus, we aim to determine the independent association between HIV infection and RH. Additionally, we will investigate the impact of other potential drivers of RH such as hypercholesteremia, obesity, chronic kidney disease, diabetes, ART regimen and duration, alcohol use as well as smoking. Understanding whether HIV increases the risk of treatment-resistant hypertension, and the influence of other potential drivers of RH is vital in targeting early optimal preventive and therapeutic interventions in this vulnerable sub-population.

For peer review only

METHODS AND ANALYSIS

Study design and setting

A case-control study will be conducted at the outpatient hypertension clinic at Queen Elizabeth Central Hospital (QECH) in Blantyre, Malawi. QECH is the largest tertiary hospital in Malawi that receives referrals from across the country. It is a free public hospital hence it serves mainly the low to middle-income class. A case-control study design has been chosen for three reasons. Firstly, treatment-resistant hypertension is a relatively rare disease, and a case-control study will allow research on this outcome. Secondly, it will reduce the time constraint that a prospective cohort study would present due to the need for a longer follow-up period. Lastly. It will allow the investigation of multiple factors associated with RH.

Identifying cases and controls

Cases will be defined as hypertensive patients with a confirmed diagnosis of treatment-resistant hypertension. In this study, treatment-resistant hypertension will be defined as a blood pressure of ≥ 140/90mmHg measured using an ambulatory blood pressure monitor (ABPM) over 4 hours in patients with hypertension and who are on at least 3 different medications including a diuretic. The 4-hour ABPM will be adopted in this study since previous studies have shown and validated a 4-hour ABPM as an accurate proxy of daytime (24 hours) blood pressure.[12] Controls will be defined as hypertensive patients with well-controlled blood pressure (BP <140/90mmHg) regardless of how many antihypertensive medications they are currently taking. For each case, two controls, frequency matched for age group and sex, will be selected from among hospital clients attending the same hypertension

For peer review only

clinic as the case (i.e., from the population giving rise to the case). The study period is from January 2022 to August 2023, with recruitment expected to conclude in June 2023. Patients who are 18 years and above presenting to the hypertension clinic at Queen Elizabeth Central Hospital will be considered for recruitment into the study. All patients presenting to the clinic with uncontrolled BP, and on at least 3 different medications including a diuretic, will be considered as potential cases, and screened for potential recruitment as outlined in Figure 1. Controls will be selected, using simple random sampling, from among a cohort of patients presenting to the same clinic, but without RH. The selection of both controls and cases will be done before the ascertainment of HIV status. Additionally, selecting controls from a population that would be giving rise to the cases as well as the use of simple random sampling will minimise any potential selection bias.

For peer review only

Recruitment

Suspected RH cases will be patients who have elevated blood pressure of ≥140/90mmHg on the day of recruitment and who are taking any three classes of antihypertensive medication including a diuretic. The investigators will measure the blood pressure of both cases and controls using a digital blood pressure cuff. After 30 minutes of the participants' rest and whilst the participant is seated, the investigators will collect 3 blood pressure readings 5 minutes apart. This method was used in previous studies to measure blood pressure in Malawian adults.[11] For participants who have pseudo-resistant hypertension (defined as elevated blood pressure ≥140/90mmHg on at least 3 medications including a diuretic, but where there is evidence of white coat hypertension, or where the cause of the uncontrolled

blood pressure is known)[9] ABMP will be used (as illustrated in figure 1 below). As part of the exclusion of pseudo-RH, the investigators will assess if the dosing of medications is correct and drug combination is not sub-optimal using the Malawi Standard Treatment Guidelines. Additionally, adherence to medications will be assessed through the Medication Adherence Scale which has been validated the African cohorts.Click or tap here to enter text.[13,14] Thereafter, those with correct doses and optimum drug combinations, who are adherent to their medication will be assessed for white coat hypertension (or lack thereof) by taking blood pressure measurements continuously over 4 hours using an ABPM. In this 4-hour period, the ABPM machine will continuously monitor blood pressure, but it will automatically record blood pressure every 30 minutes. If a patient still has a blood pressure reading of ≥140/90mmHg after this time, they will be defined as having resistant hypertension and enrolled into the study as a case.

The controls will be sex and age-group-matched individuals who have well-controlled blood pressure (Bp<140/90mmHg). After obtaining consent in both groups, HIV status will be ascertained. Other factors such as chronic kidney disease, obesity, hypercholesteremia, diabetes, smoking, alcohol use, ART regimen and duration will be assessed through the collection of medical history, review of notes in health passport books, and blood tests.

Inclusion criteria for cases

- Non-pregnant adults (≥18 years)

For peer review only

- Patients taking at least 3 classes of antihypertensive medication including a diuretic for at least a month.
- Patients with high blood pressure readings of ≥ 140/90mmHg on the last 2 hospital visits.
- Patients with a blood pressure reading of ≥ 140/90mmHg on the date of enrollment.

Exclusion criteria for cases

- Patients over 18 years with blood pressure readings of ≥ 140/90mmHg who are taking no more than two classes of antihypertensive medication.
- Patients taking at least 3 classes of antihypertensive medication but not including a diuretic on their drug regimen.

Inclusion criteria for controls

- Non-pregnant adults (≥18 years) who have well-controlled blood pressure (BP < 140/90mmHg) taking any classes of antihypertensive medication.
- Patients must have had well-controlled blood pressure (BP<140/90mmHg) on their last 2 hospital visits.

Exclusion criteria for controls

- Patients over 18 years with blood pressure readings of ≥140/90mmHg

For peer review only

- Patients over 18 years with well-controlled blood pressure (BP<140/90mmHg), who are not taking any antihypertensive medication.

Data collection

Details of the data to be that will be collected are outlined in Table 1. The research staff will first collect demographic data from all participants. Thereafter, participants will be asked about their medical and social history. For this study, information on previous diagnoses of hypertension, HIV, kidney disease, diabetes and high cholesterol will be collected. Information including duration of illness and dose of medication will also be sought. Lastly, body mass index (BMI) will be calculated and categorized by age, sex and percentile.

Objective 1: *Assessing the association between HIV infection and RH*

In a private environment, participants will first be asked if they are aware of their HIV status. HIV status will be deemed positive if the individual is on ART or has a documented positive result in their health passport. Those who have been taking ART will provide more information on regimen(s) and how long they have been taking the drugs. This includes asking if they have been switched on ART previously.

Individuals with documented negative HIV test results in their health passports within the last three months will be marked as negative. Three months have been employed in tandem with the window period for HIV infection. Rapid antibody tests (which is what will be used in this study) can accurately detect HIV infection 90 days

For peer review only

after exposure. An HIV test following the testing algorithm that is stipulated in the Malawi HIV Clinical Guidelines[15] will be offered after relevant informed consent and pre-test counselling to those who are uncertain about their status or who verbally indicate that they have a negative result without documentation in their health passport (but are willing to have their HIV status confirmed). Post-test counselling will be offered, and if found positive, participants will be referred for ART initiation. The HIV test will be done by trained providers at the Accidents and Emergency department at QECH.

Objective 2: ***Identifying other independent risk factors or predictors of RH***

In previous studies,[7,8,16] it has been shown that clinical conditions including chronic kidney disease, obesity and dyslipidaemia are associated with a higher risk of RH. In this study, these three conditions will be studied as (modifiable) risk factors. Screening for the presence of these comorbidities is part of the standard of care in patients in hypertensive patients. In circumstances where recent (<3 months) blood results to monitor for these conditions are not available, blood samples will be collected to conduct two biochemistry tests: creatinine and total cholesterol from both cases and controls. The creatinine results will be used to calculate the estimated Glomerular Filtration Rate (eGFR) that will determine the presence of chronic kidney disease. 5mls of blood will be drawn in a red top test tube to conduct the aforementioned tests. These tests will be conducted at the Malawi-Liverpool-Wellcome Research Programme's laboratory. Additionally, the weight and height of clients will be measured to calculate the body mass index. Furthermore, information

For peer review only

on diabetes, smoking, alcohol use, ART regimen and duration (for those living with HIV) will be collected through participant interviews and from hospital records.

Primary outcome

The primary outcome of the study is the adjusted odds ratio, with a 95% confidence interval, of the association between RH and HIV. This will be defined as the odds of HIV in RH patients divided by the odds of HIV in hypertensive patients without RH.

Secondary outcomes

The secondary outcome measures are the odds ratios, with 95% confidence intervals, of the independent association between the covariates representing other potential risk factors; cholesterol, obesity, kidney disease; and RH.

Sample size

In calculating the sample size, a three-fold relative difference in the odds of HIV infection in cases compared with controls was considered clinically significant. Assuming an HIV prevalence of 10.6% among controls, as is seen in the general population,[10] a sample size of 66 cases and 132 controls (a total of 198) will provide at least 80% power, at a 5% level of significance, to detect a three-fold relative difference in the odds of exposure (HIV infection) between those with- and without RH (i.e., the odds ratio of HIV infection in cases relative to controls = 3). These computations were conducted using STATA 17, using the function of estimating sample size for a matched case-control study.

For peer review only

Data management

All data in the study will be collected and managed using the Open-Data Kit software platform. Data will be entered by the study team, and the data accuracy will be verified by the study principal investigator (PI). Only study team members will have access to protected health information. All study-related information will be stored securely at the study site. Tablet computers used to collect the data will be password protected. Any physical participant information will be stored in locked file cabinets in areas with limited access. Patients will not be identified by name in any reports on this study. The study PI and co-investigators will have access to the final dataset of the study.

Data analysis plan

The number and percentages of cases and controls identified, and those recruited into the study and participants included in the final analysis will be reported. Additionally, the median (range) of baseline continuous variables (age, weight, height, body mass index, blood pressure, total cholesterol, and creatinine values in both groups) will be described for both cases and controls. Furthermore, the prevalence of other risk factors; HIV, obesity, hypercholesteremia and chronic kidney disease in both RH cases and non-RH controls, will be described. In participants living with HIV, different ART regimens and documented viral load and/or CD4 counts, in both controls and cases, will be compared.

For peer review only

To determine the primary and secondary outcomes, two main steps will be carried out. Firstly, the crude odds ratios of the association between RH and exposure characteristics of interest: HIV, obesity, chronic kidney disease, and hypercholesteremia; will be computed in univariate analysis with conditional logistic regression models. Similarly, the relationships between other exposure variables; smoking, alcohol intake and diabetes; and RH will be established. In participants living with HIV, we will explore the relationship between the different ART regimens and RH. Point estimates, their confidence intervals and statistical significance, assessed at a 5% level of significance, will be used to determine the strength of the association. Except for HIV, only covariates that show narrow confidence intervals in this univariate analysis will be carried on to the next step.

Secondly, using a backward stepwise approach in a conditional logistic regression model, the adjusted association between RH and HIV will be ascertained. Covariates to be adjusted for will be those that show a narrow confidence interval for the crude odds ratio in the first step above. For all analyses, the $\chi 2$ test will be used to compare categorical data between cases and controls, while the Wilcoxon Rank Sum test will be used for continuous data. Reporting of results will be in line with the STROBE guidelines.[17,18] All analyses will be conducted using STATA 17 software. In a sensitivity analysis, directed acyclic graphs (DAG), as previously described,[19,20] will be used to evaluate potential predictors of RH or confounders of the association between RH and HIV. Where there is a discrepancy between the identified confounders in the DAGs and the backward stepwise method, covariates identified using DAGs will be carried forward. The use of the two methods in determining confounders or other independent predictors to include in the final model

For peer review only

has been opted to ensure robustness in making causal inferences in the association between RH and HIV.

Patient and public involvement statement

Patients and the public were not involved in setting the research priorities, defining the research question, or determining the outcomes of this study.

ETHICS AND DISSEMINATION

This study has been approved by the College of Medicine Research Ethics Committee (COMREC) with approval number P.05/22/3637. Written consent will be sought from participants before enrolment into the study. Findings from this study will be disseminated through submission for publication in an international, open-access, peer-reviewed journal. Anonymised data from the study will be made available upon request from the authors.

For peer review only

REFERENCES

1 Bigna JJ, Noubiap JJ. The rising burden of non-communicable diseases in sub-Saharan Africa. Lancet Glob Health. 2019;**7**:e1295–6. doi:10.1016/S2214-109X(19)30370-5

2 van Zoest RA, van den Born BJH, Reiss P. Hypertension in people living with HIV. Curr Opin HIV AIDS. 2017;**12**:513–22. doi:10.1097/COH.0000000000000406

3 Xu Y, Chen X, Wang K. Global prevalence of hypertension among people living with HIV: a systematic review and meta-analysis. Journal of the American Society of Hypertension. 2017;**11**:530–40. doi:10.1016/j.jash.2017.06.004

4 Tenorio AR, Zheng Y, Bosch RJ, *et al.* Soluble markers of inflammation and coagulation but not T-cell activation predict non-AIDS-defining morbid events during suppressive antiretroviral treatment. *Journal of Infectious Diseases* 2014;**210**:1248–59. doi:10.1093/infdis/jiu254

5 Fahme SA, Bloomfield GS, Peck R. Hypertension in HIV-infected adults: Novel pathophysiologic mechanisms. Hypertension. 2018;**72**:44–55. doi:10.1161/HYPERTENSIONAHA.118.10893

6 Ataklte F, Erqou S, Kaptoge S, *et al.* The burden of undiagnosed hypertension in sub-Saharan Africa: a systematic review and meta analysis. *Hypertension* 2014;**16**. doi:10.1161/HYPERTENSIONAHA

7 Myat A, Redwood SR, Qureshi AC, *et al.* Resistant hypertension. BMJ (Online). 2012;**345**. doi:10.1136/bmj.e7473

For peer review only

8 Nansseu JRN, Noubiap JJN, Mengnjo MK, *et al.* The highly neglected burden of resistant hypertension in Africa: a systematic review and meta-analysis. *BMJ Open* 2016;**6**:e011452. doi:10.1136/bmjopen-2016-011452

9 Noubiap JJ, Nansseu JR, Nyaga UF, *et al.* Global prevalence of resistant hypertension: A meta-analysis of data from 3.2 million patients. Heart. 2019;**105**:98–105. doi:10.1136/heartjnl-2018-313599

10 Malawi Population-based HIV Impact Assessment (MPHIA. http://phia.icap.columbia.edu

11 Price AJ, Crampin AC, Amberbir A, *et al.* Prevalence of obesity, hypertension, and diabetes, and cascade of care in sub-Saharan Africa: a cross-sectional, population-based study in rural and urban Malawi. *Lancet Diabetes Endocrinol* 2018;**6**:208–22. doi:10.1016/S2213-8587(17)30432-1

12 Chanudet Xavier, Chau Phong Nguyen, Larroque Pierre. Short-term representatives of daytime and night-time ambulatory blood pressures. *J Hypertens* 1992;**10**:595–600.

13 Morisky DE, Ang A, Krousel-Wood M, *et al.* Predictive validity of a medication adherence measure in an outpatient setting. *J Clin Hypertens* 2008;**10**:348–54.https://onlinelibrary.wiley.com/doi/full/10.1111/j.1751-7176.2008.07572.x (accessed 9 Oct 2022).

14 Tandon S, Chew M, Eklu-Gadegbeku CK, *et al.* Validation and psychometric properties of the 8-item Morisky Medication Adherence Scale (MMAS-8) in Type 2 diabetes patients in sub-Saharan Africa. *Diabetes Res Clin Pract* 2015;**110**:129–36. doi:10.1016/j.diabres.2015.10.001

For peer review only

15 Ministry of Health and Population. Clinical Management of HIV in children and adults. Lilongwe: 2022. www.hiv.health.gov.mw

16 Lotufo PA, Pereira AC, Vasconcellos PS, *et al.* Resistant hypertension: Risk factors, subclinical atherosclerosis, and comorbidities among adults-the brazilian longitudinal study of adult health (ELSA-Brasil). *J Clin Hypertens* 2015;**17**:74–80. doi:10.1111/jch.12433

17 Vandenbroucke JP, Von Elm E, Altman DG, *et al.* Strengthening the Reporting of Observational Studies in Epidemiology (STROBE): Explanation and elaboration. Epidemiology. 2007;**18**:805–35. doi:10.1097/EDE.0b013e3181577511

18 STROBE. Strengthening the Reporting of Observational Studies in Epidemiology . 2023. https://www.strobe-statement.org/contributors/ (accessed 30 Mar 2023).

19 Greenland S, Pearl J, Robins JM. Causal Diagrams for Epidemiologic Research. *Epidemiology* 1999;**10**.https://journals.lww.com/epidem/Fulltext/1999/01000/Causal_Diagrams_for_Epidemiologic_Research.8.aspx

20 Suttorp MM, Siegerink B, Jager KJ, *et al.* Graphical presentation of confounding in directed acyclic graphs. *Nephrology Dialysis Transplantation* 2015;**30**:1418–23. doi:10.1093/ndt/gfu325

For peer review only

For peer review only

TABLES

Table 1. Details of data to be collected

	Enrolment
**Baseline characteristics**	
Name	X
Age	X
Sex	X
Phone Number	X
**Social history**	
Alcohol intake	X
Smoking	X
**Medical history**	
HIV	X
Hypertension	X
Kidney disease	X
Hypercholesteremia	X
Diabetes	X
Other medical diagnoses	X
**Anthropometric measurements**	
Height	X
Weight	X
**Blood pressure measurement**	
Systolic blood pressure	X
Diastolic blood pressure	X
4-hour ambulatory blood pressure monitoring	X
**Blood results**	
Creatinine	X
Total cholesterol	X
HIV rapid test	X

For peer review only

FIGURE AND TABLE LEGENDS

Table 1: Details of data to be collected

Figure 1. Description of study design and profile

AUTHORS STATEMENT.

JG and CGB conceptualized the research question. JG drafted the protocol manuscript. MN, FK and CGB reviewed the protocol manuscript. JG and MN will have oversight of data collection with supervision from FK and CGB.

FUNDING

This work will be supported through a Pre-MSc Fellowship Grant to JG; grant number WTT803/JG; awarded by the Joint Kamuzu University of Health Sciences and the Malawi-Liverpool-Wellcome Research Programme Training Committee with Core Training Support from the Wellcome.

CONFLICT OF INTERESTS

None

WORD COUNT

2751

For peer review only

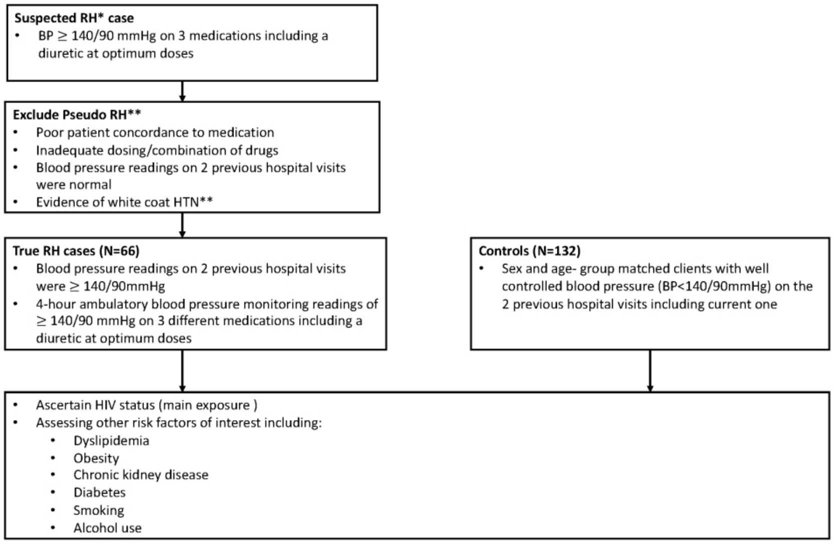

Figure 1. Description of study design and profile.
* Treatment-resistant hypertension
** Hypertension

338x190mm (200 x 200 DPI)
